# Paraquat and Parkinson’s disease: a systematic review protocol according to the OHAT approach for hazard identification

**DOI:** 10.1186/s13643-017-0491-x

**Published:** 2017-05-15

**Authors:** Carolina Vaccari, Regina El Dib, João Lauro V. de Camargo

**Affiliations:** 1 São Paulo State University (UNESP), Botucatu Medical School, Department of Pathology, Center for the Evaluation of the Environmental Impact on Human Health (TOXICAM), Botucatu, SP Brazil; 2 São Paulo State University (UNESP), Institute of Science and Technology, Department of Biosciences and Oral Diagnosis, São José dos Campos, SP Brazil; 3 McMaster Institute of Urology, McMaster University, St. Joseph’s Healthcare, Hamilton, Canada

**Keywords:** Paraquat, Parkinson’s disease, Systematic review, OHAT

## Abstract

**Background:**

Parkinson’s disease (PD) is a progressive neurodegenerative condition that has genetic susceptibility, aging, and exposure to certain chemicals as risk factors. In recent decades, epidemiological and experimental studies have investigated the role of pesticides in the development of PD, in particular that of the herbicide paraquat. Here, we, therefore, aim to systematically review the association between paraquat exposure and PD.

**Methods:**

Observational studies (cohort, case–control, and cross-sectional) eligible for this systematic review will enroll any participant who was occupationally and/or environmentally exposed to paraquat. Experimental studies, including in vivo and in vitro assays designed to assess neurotoxicological endpoints or mechanisms of paraquat neurotoxicity, will also be eligible. Outcomes of interest include the following: PD diagnosis; neurobehavioral, biochemical, and/or morphological alterations; and cellular, biochemical, and/or molecular pathways to oxidative stress. Using terms to include all forms of paraquat combined with PD, the following electronic databases will be searched: PubMed, EMBASE, LILACS, Toxnet, and Web of Science, without restrictions as to language, year, or status of publication. A team of reviewers will independently select potential titles and abstracts, extract data, assess risk of bias, and determine the overall quality of evidence for each outcome using the Office of Health Assessment and Translation (OHAT) approach for systematic reviews and evidence integration. Dichotomous data will be summarized as odds ratios, and continuous data will be given as mean differences, both with their respective 95% confidence intervals.

**Discussion:**

This is the first time that the OHAT systematic review protocol will be applied to investigate a possible causal association between exposure to paraquat and PD. Results from this study could serve as basis for regulatory agencies to define paraquat levels of concern, supporting its risk assessment process.

**Systematic review registration:**

PROSPERO CRD42016050861

**Electronic supplementary material:**

The online version of this article (doi:10.1186/s13643-017-0491-x) contains supplementary material, which is available to authorized users.

## Background

Parkinson’s disease (PD) is a progressive neurodegenerative condition characterized by selective degeneration and death of dopaminergic neurons in the *substantia nigra pars compacta* (SNpc), associated to aggregation and deposition of specific proteins (e.g., α-synucleins), oxidative stress, mitochondrial and proteasomal dysfunction, and apoptosis [1–5].

The cause of PD is not well established, but it is suggested that it has a multifactorial pathogenesis involving genetic susceptibility, aging, and possibly exposure to certain chemicals [6, 7]. In 1976, with the discovery of the *N*-methyl-4-phenyl-1,2,3,6-tetrahydropyridine molecule (MPTP), the relationship between the exposure to a neurotoxin and PD could be established. MPTP was the unintentional by-product of the meperedine derivative drug 4-propyloxy-4-phenyl-*N*-methylpiperidine (MPPP). Ever since users of MPPP-contaminated batches with MPTP developed levodopa-responsive parkinsonian symptoms, such as muteness, severe rigidity, weakness, tremor, and flat facial expression [8], this molecule has been used to induce PD in experimental models and has been useful to establish therapeutic targets [9–11].

Due to the high structural similarity between MPTP and some pesticides, in particular paraquat, a flag of concern has been raised regarding the exposure to this pesticide and the development of PD [12–16].

Paraquat (1,1′-dimethyl-4,4′-bipyridine), an important member of the bipyridylium family of broad spectrum herbicides, is commonly used to control pests in several crops, such as soybeans, sorghum, sugar cane, cotton, corn, apple, among others [17]. It interferes with photosynthetic electron transport, reducing oxygen to superoxide subsequent leading to membrane rupture and desiccation of leaves [18].

Many countries have already banned paraquat due to its acute pulmonary and cutaneous toxicity, while others have established restrictive use measures, such as limited concentrations of the active ingredient in formulated products and manipulation only by licensed mixers and ground applicators [19].

In experimental models, paraquat has also been linked to the production of reactive oxygen species (ROS), oxidative stress, and aggregation of α-synucleins in dopaminergic neurons [20, 21]. The mechanism used by paraquat to access dopaminergic neurons is not yet fully understood [13, 22].

Over the past decades, several epidemiological studies have suggested an association between exposure to pesticides as well as other environmental factors such as rural living, farming, and well water consumption, with an increased risk for PD development [23–25].

It is important to note that any epidemiological study has potential biases and confounding factors that make it difficult for result interpretation [26]. The main sources of heterogeneity detected among the observational studies are the differences in design, case and control selection, sample size, lack of satisfactory information regarding the definition, extent and duration of exposure, the non-specification of the pesticides used, as well as the accuracy of the PD diagnosis [26].

Therefore, it has not been possible to assume causality between paraquat exposure and PD based only on epidemiological studies [27, 28]. The available systematic reviews (SR) and meta-analysis were able to detect a positive association between pesticide exposure and PD [29–32]. However, few are the SR that addressed specific pesticides, such as paraquat [33–39]; due to methodological flaws and lack of statistical power in the included studies, the currently available weight of evidence is insufficient to conclude that there is a causal relationship for any particular pesticide compound and PD development [33–39].

When the evidence on the causal role of an environmental contaminant is not strong or consistent enough, other relevant information are crucial for a causal association to be considered, such as information provided by experimental animal studies and/or mechanistic studies. In this context, experts, such as the Office of Health Assessment and Translation (OHAT) team, have developed new SR protocols to better address environmental health questions, including not only observational studies, but also experimental in vivo and in vitro studies [40, 41]. Therefore, we propose to systematically review reports on the association between exposure to paraquat and PD using the OHAT approach in order to integrate human, animal, and mechanistic data [41].

Science faces a delicate moment regarding methodological flaws, inaccurate statistics, and lack of information that compromise the reproducibility of scientific studies [42]. Since regulatory decisions should be supported by science-generated evidences, it is extremely important that a comprehensive literature-based evaluation is conducted to integrate all of the available evidence regarding exposure to paraquat and PD.

## Methods and analysis

### Standards

The methods described by the Office of Health Assessment and Translation (OHAT) will be followed to conduct this review [41]. Our reporting adheres to the Preferred Reporting Items for Systematic Reviews and Meta-Analysis protocols (PRISMA-P) statement [43] and Meta-analysis of Observational Studies in Epidemiology (MOOSE) statements [44] (see Additional file 1).

### Protocol registration number

This protocol is registered in the International Prospective Register of Systematic Reviews (PROSPERO CRD42016050861).

### Eligibility criteria

As we will summarize the evidence and assess its certainty separately for bodies of evidence from observational, experimental, and mechanistic studies, we decided to present the eligibility criteria according to the type of evidence. The following criteria will be applied for observational studies:

- Type of study: Case–control, cohort, and cross-sectional studies.
- Participants: Any adult (≥18 years) identified as occupationally and/or environmentally exposed to paraquat, regardless gender.
- Exposure: Any period, frequency and amount of exposure to paraquat.
- Controls: Participants that were not exposed to paraquat.
- Primary outcomes: PD diagnosed by experts through clinical assessments and/or differential diagnosis.
- Secondary outcomes: Signs and symptoms of parkinsonism, such as tremor, bradykinesia, rigidity, postural instability, mobility, activities of daily living, emotional well-being, and cognition functions reported by the participants in interviews and/or questionnaires.

The following criteria will be applied for experimental animal studies:

- Type of study: Any study using laboratory rodents designed to assess endpoints of paraquat neurotoxicity. Studies evaluating the neuroprotective effect of a given substance in response to paraquat will not be considered.
- Animals: Rats and mice with no restrictions as to strain, gender, age, and life stage at paraquat exposure or outcome assessments.
- Exposure: Only multiple-dose non-acute studies will be considered. There will be no restrictions regarding dose or exposure route. No mixtures will be allowed.
- Controls: Animals not exposed to paraquat or any other chemical. The doses used in each study will also be considered mutual controls (dose–response).
- Primary outcomes: Morphological alterations (reduction in the average volume of SNpc, dead dopaminergic neurons in the nigrostriatal pathway and/or intracytoplasmic presence of Lewy bodies) and biochemical alterations (reduction in the levels of dopamine and its metabolites in the striatum).
- Secondary outcomes: Neurobehavioral alterations (low motor activity, low sensorimotor reflexes, effects as to cognitive function) and detection of reactive oxygen species—ROS—in dopaminergic neurons.

The following criteria will be applied for mechanistic studies:

- Type of study: In vivo studies with rodents and in vitro studies designed to assess cellular, biochemical, and/or molecular mechanisms of paraquat neurotoxicity.
- Animals (for in vivo studies): Rats and mice with no restrictions as to strain, gender, age, and life stage at exposure or outcome assessments.
- Cell line (for in vitro studies): Cell lines such as SK-N-SH, SH-SY5Y, PC12, RBE, astrocytes, dopaminergic neurons, or other cell lines used in in vitro models for PD.
- Controls (for in vitro and in vivo studies): Systems not exposed to paraquat. The doses used in each study will also be considered mutual controls (dose–response).
- Exposure (for in vitro and in vivo studies): For in vivo studies, criteria adopted for experimental rodent studies will be applied. For in vitro studies, the criteria adopted for conventional in vitro studies will be applied, for example, doses should not reach cytotoxicity levels. No mixtures will be allowed.
- Outcomes (for in vitro and in vivo studies): Cellular, biochemical, and/or molecular pathways to death of dopaminergic neurons, such as oxidative stress, mitochondrial dysfunction, intracytoplasmic presence of Lewy bodies in dopaminergic neurons, and/or other key molecular initiating events related to parkinsonism.

### Search methods for primary studies

#### Electronic searches

Peer-reviewed original studies published in the following electronic databases will be searched: PubMed (1966 to present), EMBASE (1980 to present), Web of Science (1990 to present), Toxnet (2007 to present), and LILACS (1982 to present), without language, year, and status of publication restrictions.

### Search strategy

MeSH terms and free terms related to “Parkinson’s Disease,” “herbicides,” and “paraquat” will be combined. The search strategy will be adapted for each database (Table 1).

**Table 1:** Search strategy

(Idiopathic Parkinson’s Disease OR Lewy Body Parkinson Disease OR Lewy Body Parkinson’s Disease OR Primary Parkinsonism OR Idiopathic Parkinson Disease OR Parkinson’s Disease OR Parkinson Disease OR Parkinson Patients OR Parkinson Patient OR Parkinson’s Patients OR Parkinson’s Patient OR Paralysis Agitans) AND (Herbicides OR Methyl Viologen OR Gramoxone OR Paragreen A OR paraquat OR paraquat poisoning OR paraquat poisoned patients OR paraquat poisoned patient OR paraquat intoxication OR paraquat concentration OR neurotoxicity of paraquat OR paraquat neurotoxicity).	

References listed in the selected studies will be analyzed for additional citations. Studies’ main authors will eventually be contacted for potentially missing data.

### Searching other resources

Screening of the “grey literature” will also be performed to identify publications not commercially published or not readily available to the public. Examples of “grey literature” include technical reports from scientific research groups or government agencies, such as US Environmental Protection Agency (EPA), Food and Drug Administration (FDA), Agency for Toxic Substances and Disease Registry (ATSDR), and National Institute for Occupational Safety and Health (NIOSH).

### Eligibility determination

Two reviewers (CV and JLVC) will independently screen all titles and abstracts identified by the literature search, obtain and read full-text articles of all potentially eligible studies, and evaluate them for eligibility. Disagreements will be resolved by consensus and, eventually, through consultation with technical advisors to improve accuracy and consistency among screeners.

### Study flow diagram

A PRISMA flow diagram will be produced to indicate the number of included and excluded studies and the corresponding reasons for exclusion.

### Data extraction

Reviewers will undergo calibration exercises and work in pairs to independently extract data from included studies. A standard form will be used to extract information from the included observational studies, such as study design, study characteristics (e.g., sample sizes, number of cases, variables for which controls were matched to cases, study population, and confounding factors), type of exposure (i.e., occupational, environmental, or both), details on paraquat exposure (e.g., source, duration, frequency, intensity), methods of exposure measurement (e.g., questionnaires/interviews or fluid analysis such as blood and urine for characterization of possible internal exposure), criteria used for PD diagnosis, effect estimate value or description of qualitative results, etc.

Data extracted from experimental animal studies will include experimental design, guideline compliance (e.g., use of EPA (Environmental Protection Agency), OECD (Organization for Economic Co-operation and Development), NTP (National Toxicology Program), or another guideline for study design), animal characteristics (specie, strain, gender, genetic background, number of groups, number of animals per group, age, or life stage at start of dosing and at health outcome assessment), paraquat characteristics (supplier, catalog number, and purity), treatment (doses, frequency, eventual information on internal dosimetry), vehicle used, routes of administration, protocol (randomization procedure, evaluated outcome(s), methods for assessing outcomes(s), blinding during outcome assessment, statistical methods), results (measures of effect at each dose or concentration level (e.g., mean, median, frequency, and measures of precision or variance) or description of qualitative results, no observed effect level (NOEL), lowest observed effect level (LOEL), benchmark dose (BMD) analysis), etc.

For in vitro studies, the following information will be extracted: cell/tissue model (cell line, cell type or tissue, source of cells/tissue,sex of human/animal of origin, specie, strain), name and source of assay kits, treatment (concentration levels, as presented and converted to micromolar when possible, duration, and frequency of dosing), protocol description (number of replicates per group, randomization procedure), outcome (outcome(s) assessed, methods for assessment, blinding during outcome assessment), results (NOEC (no observed effect concentration), LOEC (lowest observed effect concentration), IC50 (inhibitory concentration 50), statistical methods, etc.

### Assessment of internal validity of individual studies

Reviewers, working in pairs, will independently assess the risk of bias of each human or animal study; a specific protocol to address the risk of bias of mechanistic studies is still in development. Possible sources of bias will be assessed through 11 pre-defined questions considering (1) potential confounders, (2) confidence in exposure characterization, and (3) confidence in the outcome assessment [45]. The answer to each question is assigned to one of the four following categories of risk of bias: definitely low, probably low, probably high, or definitely high. After assigning each response to one of the categories, we will also use a three-tier system to classify the studies regarding their overall methodological quality. Studies considered at definitely or probably low risk of bias will be grouped as tier 1 studies. Tier 3 studies refer to the ones considered to be at definitely or probably high risk of bias; tier 2 will encompass studies that did not meet the criteria for tier 1 and tier 3 and therefore fall into an in-between category [41].

### Data synthesis and statistical analysis

Meta-analysis of results will be considered by investigating heterogeneity among animal and human studies, separately. It is common, though, that environmental health studies have some differences regarding outcome assessments and exposure definitions that could be an obstacle to formal statistical meta-analysis [26]. The heterogeneity associated with pooled effect estimates will be assessed with the use of a *χ* ^2^ test and the *I* ^2^ statistic (*I* ^2^ = [(Q − df)/Q] × 100%) [46, 47]. Heterogeneity will be classified as follows: 0 to 40% (no important heterogeneity); 30 to 60% (moderate heterogeneity); 50 to 90% (substantial heterogeneity); and 75 to 100% (considerable heterogeneity).

Statistical analysis will be conducted using the Comprehensive Meta-Analysis STATA software (version 10.1). If considerable heterogeneity is detected among studies, meta-analysis will not be indicated and results will only be presented in tables or in a narrative synthesis. However, if heterogeneity does not exceed 75%, we will use random effects meta-analysis [48], which is a more conservative approach of pooling the results. In this case, the measure of association will be presented as odds ratios and mean difference, both with a 95% confidence interval (95% CI), for studies with dichotomous and continuous data, respectively [49].

### Subgroup and sensitivity analysis

Subgroup analysis will be conducted to investigate possible heterogeneity causes and other risk factors that could be potential cofounders. Studies will be allocated into groups according to common characteristics, as described below. The outcomes are assessed to determine if exposure caused a significant effect due to a specific feature. For human studies, subgroup analyses will be conducted for different exposure periods (<5 years vs. ≥5 years), co-exposures to other pesticides also related to PD (e.g., paraquat plus rotenone vs. paraquat plus maneb vs. only paraquat), different comorbidities (e.g., smoking vs. alcohol vs. diabetes), different age groups (e.g., <60 years of age vs. ≥60 years), and presence or absence of family history of PD.

For experimental animal studies, subgroup analyses will be conducted for dose (e.g., higher vs. lower), duration of paraquat exposure in multidose studies, and routes of administration (e.g., oral vs. dermal vs. inhalation vs. intraperitoneal vs. intravenous).

If there are enough studies, we will develop sensitivity analyses to explore robustness of the results for each type of study design “e.g., observational studies (cohort, case–control, cross-sectional) vs. experimental animal studies, cohort vs. case–control vs. cross-sectional” and studies with low risk of bias vs. those with high risk of bias.

### Certainty of evidence

The certainty of the evidence for each outcome will also be rated at high, moderate, low, or very low, using an adapted version of the Grading of Recommendations Assessment, Development and Evaluation system (GRADE) [41]. To date, this adjusted protocol was only applied to human and animal studies that evaluate toxicological outcomes and is not yet used to evaluate mechanistic studies.

Available studies on a particular outcome will be initially grouped by key study design features that guarantee that the paraquat exposure preceded and was associated with the development of the signs and symptoms of parkinsonism [41]. Each grouping of studies will be given an initial certainty rating depending on the presence of those features.

In this adaptation of GRADE, experimental studies begin as “high certainty” of the evidence, cohort studies as “moderate certainty,” case–control studies as “moderate or low certainty,” and cross-sectional studies as “low certainty” of the evidence. This initial rating could be downgraded by the presence of risk of bias, imprecision, unexplained inconsistency, indirectness, and publication bias [41]. If there is a minimum of ten studies included in the systematic review, funnel plots might be used to assess the possibility of publication biases [50]. Egger’s regression test and “trim and fill” techniques might also be used to visualize asymmetrical or symmetrical patterns of study results [51].

On the other hand, the presence of a large magnitude of effect, a dose–response gradient, recognition of confounding factors that could underestimate or overestimate the effect and consistency among studies with different designs could upgrade the certainty of the evidence [41]. After the evaluation of each study group for each outcome, only the ones with the highest level of certainty will be translated into the respective level of evidence, as indicated below.

### Translating certainty ratings into levels of evidence for parkinsonian-like effects

Five descriptors will be used to rate the level of evidence: “high,” “moderate,” “low,” “inadequate evidence,” and “evidence of no health effect.” The first three descriptors (“high,” “moderate,” and “low” level of confidence) used in the previous step to indicate the certainty of the evidence will be directly converted into levels of evidence. However, if the level of certainty is “very low” or no evidence is identified, the level of evidence will be considered “inadequate” [41].

The descriptor “evidence of no health effect” that indicates that paraquat is not related to PD in humans or in rodent models will be considered only when the level of certainty is high [41].

### Integrated evidence for paraquat hazard identification

For proper categorization of paraquat in one of the five categories of hazard (known, suspected, presumed, not classifiable, or not identified to be hazardous to humans) [41], evidence coming from human and animal studies will be integrated with mechanistic data that may be relevant to support biological plausibility and increase or decrease the hazard classification.

Among the factors that can support biological plausibility and increase the hazard classification, the magnitude of the effect, the dose–response gradient, and the direct and indirect consistencies between outcomes from studies with different biological levels (human, rodents, and in vitro) will be considered. On the contrary, hazard classification may be reduced by the identification of risk of bias, unexplained inconsistencies between studies with related outcomes, non-relevance of the paraquat mechanism of toxicity to humans and dose levels not relevant to real human exposure.

## Discussion

Current systematic review protocols are primarily based on randomized clinical trials for evaluating the efficacy of healthcare interventions [52–54]. With the increasing interest of using systematic reviews to study possible associations between exposure to environmental chemicals and the development of diseases, experts have developed new protocols to better address environmental health questions [40, 41]. The existing systematic reviews involving exposure to paraquat and PD development deal only with non-randomized observational studies [33–39]. Although these studies are fundamental in evaluating possible human health effects, their design limitations may be an obstacle for causality inferences [28]. The protocol proposed by OHAT is currently the most complete literature-based evaluation to systematically review the quality of evidence raised by studies of different biological and operational levels of complexity: epidemiologic studies with human beings, in vivo experimental animal studies with rodents, and in vitro studies with different cell lines. Ultimately, those evidences are integrated to indicate the hazard that paraquat represents to humans (causal or promotor). Many of the aspects considered by the GRADE system of evaluation resemble the causality criteria proposed by Sir Bradford Hill, or what he called “considerations” for causation, such as strength, consistency, temporality, biological plausibility, dose–response gradient, and experimental evidence [27, 41].

Results that could arise from the present study could serve as basis for regulatory agencies to define paraquat levels of concern (LoC), once the real human levels of exposure and the relevance of its mechanisms of neurotoxicity are known. Hazard identification is the early stage of the risk assessment process, which takes into account information such as LoC and exposure levels for regulatory purposes [41]. The systematization of this protocol will ensure a proper assessment of the available evidences, leading to an updated scientific judgment on the potential association between paraquat and PD.

## Additional file

Additional file 1: PRISMA-P 2015 checklist. This checklist includes a list of recommended items to include in a systematic review protocol (DOCX 36 kb).

## Abbreviations

95% CI: 95% confidence interval
ATSDR: Agency for Toxic Substances and Disease Registry
BMD: Benchmark dose
EPA: Evironmental Protection Agency
FDA: Food and Drug Administration
GRADE: Grading of Recommendations Assessment, Development and Evaluation System
IC50: Inhibitory concentration 50
LoC: Levels of concern
LOEC: Lowest observed effect concentration
LOEL: Lowest observed effect level
MPTP: *N*-Methyl-4-phenyl-1,2,3,6-tetrahydropyridine
NOEC: No observed effect concentration
NOEL: No observed effect level
NTP: National Toxicology Program
OECD: Organisation for Economic Co-operation and Development
OHAT: Office of Health Assessment and Translation
PRISMA-P: Preferred Reporting Items for Systematic Reviews and Meta-Analysis protocols
ROS: Reactive oxygen species
SNpc: *Substantia Nigra pars compacta*
SR: Systematic reviews

## References

[1] Greenamyre JT, MacKenzie G, Peng T, Stephans SE (1999). Mitochondrial dysfunction in Parkinson’s disease. Biochem Soc Symp.

[2] Jenner P (2003). Oxidative stress in Parkinson’s disease. Ann Neurol.

[3] Ebadi M, Sharma SK (2003). Peroxynitrite and mitochondrial dysfunction in the pathogenesis of Parkinson’s disease. Antioxid Redox Signal.

[4] McNaught KS, Olanow CW (2003). Proteolytic stress: a unifying concept for the etiopathogenesis of Parkinson’s disease. Ann Neurol.

[5] Alves DC (2003). Recent advances on a-synuclein cell biology: functions and dysfunctions. Curr Mol Med.

[6] Di Monte DA, Lavasani M, Manning-Bog AB (2002). Environmental factors in Parkinson’s disease. Neurotoxicology.

[7] Warner TT, Schapira AH (2003). Genetic and environmental factors in the cause of Parkinson’s disease. Ann Neurol.

[8] Langston JW, Ballard P, Tetrud JW, Irwin I (1983). Chronic Parkinsonism in humans due to a product of meperidine-analog synthesis. Sci Transl Med.

[9] He XJ, Uchida K, Megumi C, Tsuge N, Nakayama H (2015). Dietary curcumin supplementation attenuates 1-methyl-4-phenyl-1,2,3,6-tetrahydropyridine (MPTP) neurotoxicity in C57BL mice. J Toxicol Pathol.

[10] Jing H, Wang S, Wang M, Fu W, Zhang C, Xu D (2017). Isobavachalcone attenuates MPTP-induced Parkinson’s disease in mice by inhibition of microglial activation through NF-κB pathway. PLoS One.

[11] Elgueta D, Aymerich MS, Contreras F, Montoya A, Celorrio M, Rojo-Bustamante E (2017). Pharmacologic antagonism of dopamine receptor D3 attenuates neurodegeneration and motor impairment in a mouse model of Parkinson’s disease. Neuropharmacology.

[12] Fei Q, McCormack AL, Di Monte DA, Ethell DW (2002). Paraquat neurotoxicity is mediated by a Bak-dependent mechanism. J Biol Chem.

[13] Richardson JR (2005). Paraquat neurotoxicity is distinct from that of MPTP and rotenone. Toxicol Sci.

[14] Yang W, Chen L, Ding Y, Zhuang X, Kang UJ (2007). Paraquat induces dopaminergic dysfunction and proteasome impairment in DJ-1-deficient mice. Hum Mol Genet.

[15] Morán JM, Gonzalez-Polo RA, Ortiz-Ortiz MA, Niso-Santano M, Soler G, Fuentes JM (2008). Identification of genes associated with paraquat-induced toxicity in SH-SY5Y cells by PCR array focused on apoptotic pathways. J Toxicol Environ Health A.

[16] Yang W, Tiffany-Castiglioni E, Koh HC, Sona IH (2009). Paraquat activates the IRE1/ASK1/JNK cascade associated with apoptosis in human neuroblastoma SH-SY5Y cells. Toxicol Lett.

[17] Paraquat Information Center on behalf of Syngenta Crop Protection AG. http://paraquat.com/use/crops. Accessed 03 Nov 2016.

[18] Esfahani M (2012). Effects of pre-harvest application of paraquat on grain moisture reduction, grain yield and quality of rapeseed (Brassica napus L.) cultivars. Caspian J Environ Sci.

[19] ANVISA (Agência Nacional de Vigilância Sanitária). Parecer Técnico de Reavaliação [Technical report of reevaluation] n°1 GGTOX/ANVISA, 2015. http://portal.anvisa.gov.br/documents/33880/2541353/CP%2B94-2015%2B-%2BNT.pdf/50fb348f-3c2a-4992-a3a2-ca89fd4d2127. Accessed 11 Nov 2016.

[20] Kuter K, Smiałowska M, Wieronska J, Zieba B, Wardas J, Pietraszek M (2007). Toxic influence of subchronic paraquat administration on dopaminergic neurons in rats. Brain Res.

[21] Kuter K, Nowak P, Golembiowska K, Ossowska K (2010). Increased reactive oxygen species production in the brain after repeated low-dose pesticide paraquat exposure in rats: a comparison with peripheral tissues. Neurochem Res.

[22] Liou HH, Chen RC, Tsai YF, Chen WP, Chang YC, Tsai MC (1996). Effects of paraquat on the substantia nigra of the Wistar rats: neurochemical, histological, and behavioral studies. Toxicol Appl Pharmacol.

[23] Le Couteur DG, Mc Lean AJ, Taylor MC, Woodham BL, Board PG (1999). Pesticides and Parkinson’s disease. Biomed Pharmacother.

[24] Kamel F, Tanner C, Umbach D, Hoppin JA, Alavanja MCR, Blair A (2006). Pesticide exposure and self-reported Parkinson’s disease in the agricultural health study. Am J Epidemiol.

[25] Dick FD, De Palma G, Ahmadi A, Scott NW, Prescott GJ, Bennett J (2007). Environmental risk factors for Parkinson’s disease and parkinsonism: the Geoparkinson study. Occup Environ Med.

[26] Berry C, La Vecchia C, Nicotera P (2010). Paraquat and Parkinson’s disease. Cell Death Differ.

[27] Hill AB (1965). The environment and disease: association or causation?. Proc R Soc Med.

[28] Adami HO, Berry SCL, Breckenridge CB, Smith LL, Swenberg JA, Trichopoulos D (2011). Toxicology and epidemiology: improving the science with a framework for combining toxicological and epidemiological evidence to establish causal inference. Toxicol Sci.

[29] Priyadarshi A, Khuder SA, Schaub EA, Shrivastava S (2000). A meta-analysis of Parkinson’s disease and exposure to pesticides. Neurotoxicology.

[30] Priyadarshi A, Khuder SA, Schaub EA, Priyardarshi SS (2001). Environmental risk factors and Parkinson’s disease: a metaanalysis. Environ Res.

[31] Van der Mark M, Brower M, Kromhout H, Nijssen P, Huss A, Vermeulen R (2011). Is pesticide use related to Parkinson disease? Some clues to heterogeneity in study results. Environ Health Perspect.

[32] Freire C, Koifman S (2012). Pesticide exposure and Parkinson’s disease: epidemiological evidence of association. Neurotoxicology.

[33] Li AA, Mink PJ, McIntosh LJ, Teta MJ, Finley B (2005). Evaluation of epidemiologic and animal data associating pesticides with Parkinson’s disease. J Occup Environ Med.

[34] Brown TP, Rumsby PC, Capleton AC, Rushton L, Levy LS (2006). Pesticides and Parkinson’s disease—Is there a link?. Environ Health Perspect.

[35] Van Maele-Fabry G, Hoet P, Vilain F, Lison D (2012). Occupational exposure to pesticides and Parkinson’s disease: a systematic review and meta-analysis of cohort studies. Environ Int.

[36] Allen MT, Levy LS (2013). Parkinson’s disease and pesticide exposure—a new assessment. Crit Rev Toxicol.

[37] Pezzoli G, Cereda E (2013). Exposure to pesticides or solvents and risk of Parkinson disease. Neurology.

[38] Ntzani EE, Chondrogiorgi M, Nitritsos G, Tzoulaki I. Literature review on epidemiological studies linking exposure to pesticides and health effects. EFSA supporting publication:EN-497, 2013. http://onlinelibrary.wiley.com/doi/10.2903/sp.efsa.2013.EN-497/abstract. Accessed 09 May 2017.

[39] Breckenridge CB, Berry C, Chang ET, Sielken RL, Mandel JS (2016). Association between Parkinson’s disease and cigarette smoking, rural living, well-water consumption, farming and pesticide use: systematic review and meta-analysis. PLoS ONE.

[40] Koustas E, Lam J, Sutton P, Johnson PI, Atchley D, Sen S, et al. Applying the navigation guide: case study #1. The Impact of Developmental Exposure to Perfluorooctanoic Acid (PFOA) On Fetal Growth. A Systematic Review of the Non-Human Evidence-Protocol, 2013. http://www.prhe.ucsf.edu/prhe/pdfs/PFOA%20Human%20Protocol.pdf. Accessed 09 May 2017.

[41] NTP (National Toxicology Program). Handbook for conducting a literature-based health assessment using OHAT approach for systematic review and evidence integration. Office of Health Assessment and Translation (OHAT), 2015. http://ntp.niehs.nih.gov/ntp/ohat/pubs/handbookjan2015_508.pdf. Accessed 14 July 2016.

[42] Berry C (2016). The dangers of hazards. Toxicol Res.

[43] Shamseer L, Moher D, Clarke M, Ghersi D, Liberati A (deceased), Petticrew M, et al. Preferred reporting items for systematic review and meta-analysis protocols (PRISMAP) 2015: elaboration and explanation. BMJ. 2015;2:g7647. 10.1136/bmj.g7647 25555855

[44] Stroup DF, Berlin JA, Morton SC, Olkin I, Williamson GD, Rennie D (2000). Meta-analysis of observational studies in epidemiology: a proposal for reporting. JAMA.

[45] OHAT (Office of Health Assessment and Translation) (2015). OHAT risk of bias rating tool for human and animal studies.

[46] Higgins JPT, Altman DG, Sterne JAC. Assessing risk of bias in included studies. In: Higgins JPT, Green S. Cochrane Handbook for Systematic Reviews of Interventions Version 5.1.0. The Cochrane Collaboration, 2011. http://handbook.cochrane.org/. Accessed 11 Nov 2016.

[47] Higgins JP, Thompson SG, Deeks JJ, Altman DG (2003). Measuring inconsistency in meta-analyses. BMJ.

[48] Murad MH, Montori V, Ioannidis J, Prasad K, Cook DJ, Guyatt G, Guyatt G, Rennie D, Meade M, Cook D (2008). Advanced topics in systematic reviews. Fixed-effects and random-effects models. Users’ guides to the medical literature: a manual for evidence based clinical practice.

[49] Vesterinen HM, Sena ES, Egan KJ, Hirst TC, Churolov L, Currie GL (2014). Meta-analysis of data from animal studies: a practical guide. J Neurosci Methods.

[50] Sterne JA, Egger M (2001). Funnel plots for detecting bias in meta-analysis: guidelines on choice of axis. J Clin Epidemiol.

[51] Egger M, Smith GD, Schneider M (1997). Bias in meta-analysis detected by a simple, graphical test. BMJ.

[52] El Dib R, Gomaa H, Carvalho RP, Camargo SE, Bazan R, Barretti P (2016). Enzyme replacement therapy for Anderson-Fabry disease. Cochrane Database Syst Rev.

[53] McGuinness MB, Karahalios A, Finger RP, Guymer RH, Simpson JA. Age-related macular degeneration and mortality: a systematic review and meta-analysis. Ophthalmic Epidemiol. 2017;24:141–52. 10.1080/09286586.2016.1259422 28139151

[54] Wang Z, Xiao L, Guo H, Zhao G, Ma J (2017). The efficiency and safety of fibrin sealant for reducing blood loss in primary total hip arthroplasty: a systematic review and meta-analysis. Int J Surg.

